# Supplementary figures and images for: Identification of chromosome ploidy and karyotype analysis of cherries (Prunus pseudocerasus Lindl.) in Guizhou
Source: PeerJ. 2024 Dec 16;12:e18668. doi: 10.7717/peerj.18668 (PMC11657202; doi:10.7717/peerj.18668)

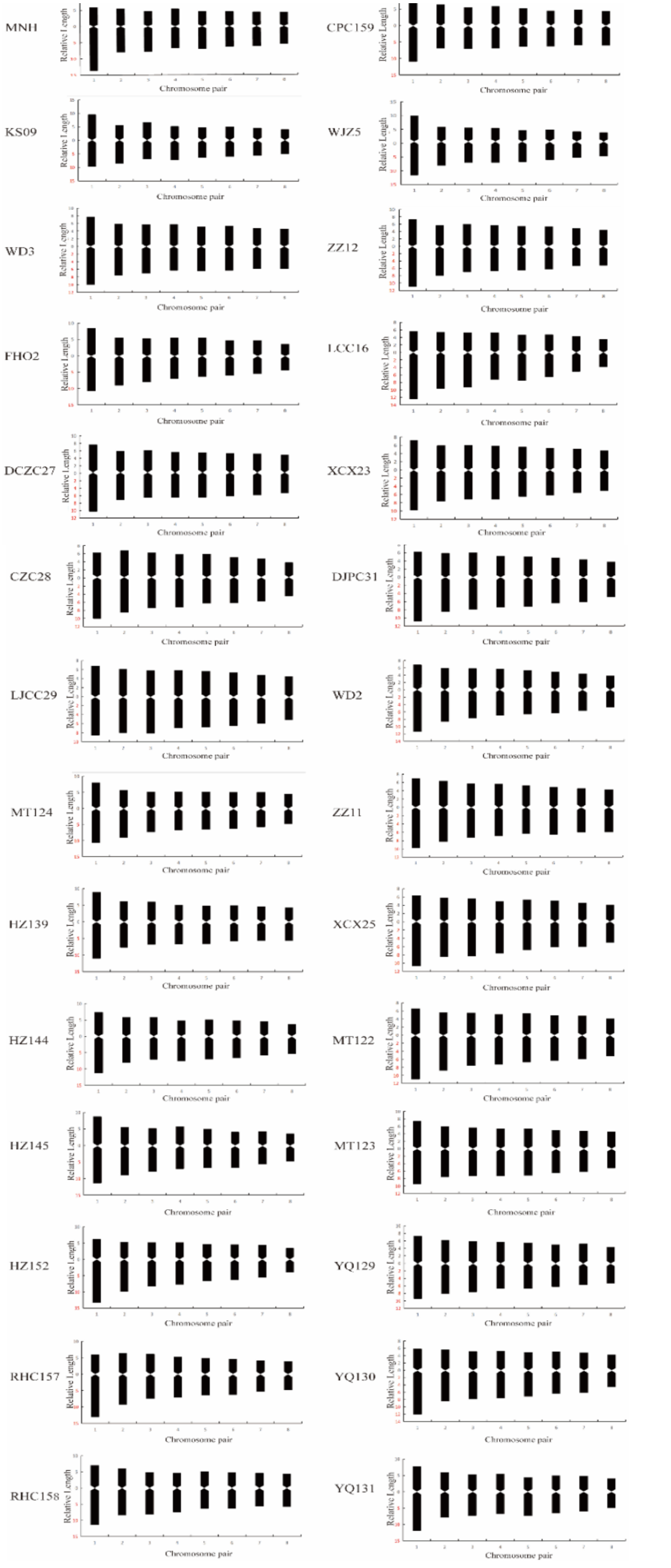

Supplement: Supplemental Information 2 [file peerj-12-18668-s002.png]

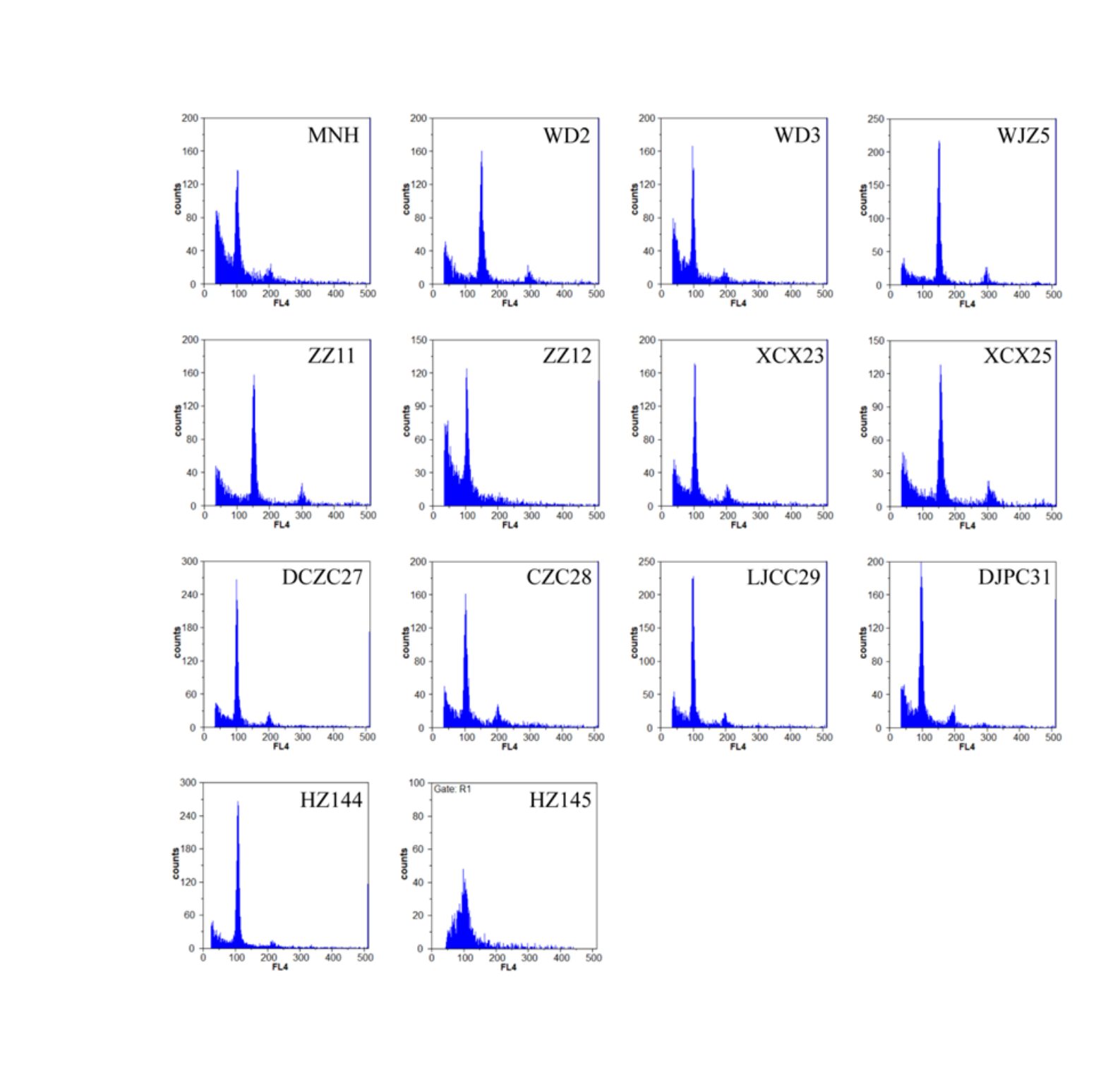

Supplement: Supplemental Information 3 — The horizontal coordinate represents the relative DNA content, and the vertical coordinate represents the number of nuclei. [file peerj-12-18668-s003.png]
