# Supplementary material for: Identification of chromosome ploidy and karyotype analysis of cherries (Prunus pseudocerasus Lindl.) in Guizhou
Source: PeerJ. 2024 Dec 16;12:e18668. doi: 10.7717/peerj.18668 (PMC11657202; doi:10.7717/peerj.18668)
Supplement: Supplemental Information 4 [file peerj-12-18668-s004.docx]

**Table S1**  The index of relative length values for each chromosome

| EMN | CN. | RL (%) | MAR | Type | ENM | CN | RL( %) | MAR | Type |
| --- | --- | --- | --- | --- | --- | --- | --- | --- | --- |
| MNH | 1 | 13.63+5.85=19.48 | 2.33 | sm | KS09 | 1 | 9.67+9.51=19.18 | 1.02 | m |
|  | 2 | 7.99+5.45=13.44 | 1.46 | m |  | 2 | 8.50+5.58=14.08 | 1.52 | m |
|  | 3 | 7.79+4.70=12.48 | 1.66 | m |  | 3 | 6.85+6.68=13.53 | 1.03 | m |
|  | 4 | 6.72+5.34=12.07 | 1.26 | m |  | 4 | 7.20+5.15=12.35 | 1.40 | m |
|  | 5 | 6.96+4.57=11.53 | 1.52 | m |  | 5 | 6.31+4.71=11.02 | 1.34 | m |
|  | 6 | 6.31+4.48=10.79 | 1.41 | m |  | 6 | 5.95+4.99=10.93 | 1.19 | m |
|  | 7 | 6.21+4.33=10.53 | 1.43 | m |  | 7 | 5.53+4.52=10.05 | 1.22 | m |
|  | 8 | 5.48+4.20=9.68 | 1.31 | m |  | 8 | 4.90+3.97=8.86 | 1.23 | m |
| WD2 | 1 | 11.35+6.80=18.15 | 1.67 | m | WD3 | 1 | 9.98+7.72=17.70 | 1.67 | m |
|  | 2 | 8.56+5.83=14.39 | 1.47 | m |  | 2 | 7.52+5.84=13.37 | 1.47 | m |
|  | 3 | 7.62+5.75=13.36 | 1.33 | m |  | 3 | 7.00+5.75=12.75 | 1.33 | m |
|  | 4 | 6.94+5.63=12.57 | 1.23 | m |  | 4 | 6.25+5.77=12.02 | 1.23 | m |
|  | 5 | 6.61+5.29=11.90 | 1.25 | m |  | 5 | 6.42+5.16=11.59 | 1.25 | m |
|  | 6 | 6.27+4.88=11.16 | 1.29 | m |  | 6 | 6.25+5.35=11.59 | 1.29 | m |
|  | 7 | 5.65+4.33=9.97 | 1.30 | m |  | 7 | 5.81+4.75=10.56 | 1.30 | m |
|  | 8 | 4.72+3.78=8.50 | 1.25 | m |  | 8 | 5.89+4.53=10.42 | 1.25 | m |
| FHO2 | 1 | 10.77+8.37=19.14 | 1.29 | m | ZZ11 | 1 | 9.74+6.96=16.69 | 1.40 | m |
|  | 2 | 9.02+5.51=14.53 | 1.64 | m |  | 2 | 8.22+6.29=14.52 | 1.31 | m |
|  | 3 | 7.95+5.27=13.22 | 1.51 | m |  | 3 | 7.23+5.71=12.94 | 1.27 | m |
|  | 4 | 7.01+5.49=12.50 | 1.28 | m |  | 4 | 6.81+5.59=12.39 | 1.22 | m |
|  | 5 | 6.35+6.35=11.88 | 1.15 | m |  | 5 | 6.25+5.25=11.50 | 1.19 | m |
|  | 6 | 6.01+4.67=10.69 | 1.29 | m |  | 6 | 6.46+4.84=11.30 | 1.33 | m |
|  | 7 | 5.44+4.64=10.08 | 1.17 | m |  | 7 | 5.94+4.53=10.47 | 1.31 | m |
|  | 8 | 4.41+3.56=7.96 | 1.24 | m |  | 8 | 5.92+4.27=10.18 | 1.39 | m |
| XCX25 | 1 | 10.61+6.23=16.84 | 1.70 | m | DCZC27 | 1 | 10.17+7.63=17.80 | 1.33 | m |
|  | 2 | 8.44+5.71=14.15 | 1.48 | m |  | 2 | 7.13+5.93=13.06 | 1.20 | m |
|  | 3 | 8.30+5.53=13.84 | 1.50 | m |  | 3 | 6.44+6.13=12.57 | 1.05 | m |
|  | 4 | 7.63+4.90=12.53 | 1.56 | m |  | 4 | 6.43+5.66 =12.09 | 1.14 | m |
|  | 5 | 6.73+5.31=12.04 | 1.27 | m |  | 5 | 6.38+5.50=11.88 | 1.16 | m |
|  | 6 | 6.02+5.06=11.08 | 1.19 | m |  | 6 | 6.15+5.32=11.46 | 1.16 | m |
|  | 7 | 6.00+4.48=10.49 | 1.34 | m |  | 7 | 5.81+5.22=11.04 | 1.11 | m |
|  | 8 | 5.00+4.02=9.02 | 1.24 | m |  | 8 | 5.24+4.86=10.10 | 1.08 | m |
| CZC28 | 1 | 10.05+6.25=16.31 | 1.61 | m | LJCC29 | 1 | 8.53+6.74=15.27 | 1.27 | m |
|  | 2 | 8.45+6.75=15.21 | 1.25 | m |  | 2 | 7.94+6.04=13.98 | 1.31 | m |
|  | 3 | 7.33+6.21=13.55 | 1.18 | m |  | 3 | 8.09+5.70=13.79 | 1.42 | m |
|  | 4 | 7.17+5.81=12.97 | 1.23 | m |  | 4 | 6.89+5.84=12.73 | 1.18 | m |
|  | 5 | 6.23+5.89=12.12 | 1.06 | m |  | 5 | 6.71+5.61=12.32 | 1.20 | m |
|  | 6 | 6.14+5.11=11.25 | 1.20 | m |  | 6 | 6.41+5.30=11.71 | 1.21 | m |
|  | 7 | 5.69+4.75=10.45 | 1.20 | m |  | 7 | 5.91+4.76=10.66 | 1.24 | m |
|  | 8 | 4.41+3.75=8.15 | 1.18 | m |  | 8 | 5.14+4.40=9.54 | 1.17 | m |
| MT122 | 1 | 10.91+6.56=17.46 | 1.66 | m | MT123 | 1 | 9.46+7.34=16.80 | 1.29 | m |
|  | 2 | 8.70+5.55=14.25 | 1.57 | m |  | 2 | 7.54+5.93=13.47 | 1.27 | m |
|  | 3 | 7.48+5.48=12.97 | 1.36 | m |  | 3 | 7.21+5.56=12.78 | 1.30 | m |
|  | 4 | 7.21+5.13=12.33 | 1.41 | m |  | 4 | 7.25+5.33=12.58 | 1.36 | m |
|  | 5 | 6.65+5.34=11.98 | 1.25 | m |  | 5 | 7.15+5.29=12.43 | 1.35 | m |
|  | 6 | 6.28+4.86=11.14 | 1.29 | m |  | 6 | 6.49+4.89=11.39 | 1.33 | m |
|  | 7 | 5.93+4.72=10.65 | 1.26 | m |  | 7 | 6.20+4.76=10.964 | 1.30 | m |
|  | 8 | 5.15+4.07=9.22 | 1.27 | m |  | 8 | 5.13+4.46=9.59 | 1.15 | m |
| MT124 | 1 | 10.60+7.98=18.58 | 1.33 | m | YQ129 | 1 | 9.35+7.20=16.55 | 1.30 | m |
|  | 2 | 9.02+5.59=14.61 | 1.61 | m |  | 2 | 8.06+6.10=14.16 | 1.32 | m |
|  | 3 | 7.32+5.05=12.37 | 1.45 | m |  | 3 | 7.56+5.87=13.42 | 1.29 | m |
|  | 4 | 6.70+5.15=11.85 | 1.30 | m |  | 4 | 6.70+5.62=12.33 | 1.19 | m |
|  | 5 | 6.57+5.05=11.62 | 1.30 | m |  | 5 | 6.67+5.38=12.05 | 1.24 | m |
|  | 6 | 6.20+4.97=11.17 | 1.25 | m |  | 6 | 6.17+4.94=11.11 | 1.25 | m |
|  | 7 | 5.72+4.95=10.68 | 1.16 | m |  | 7 | 5.65+5.15=10.80 | 1.10 | m |
|  | 8 | 4.72+4.41=9.12 | 1.07 | m |  | 8 | 5.33+4.25=9.59 | 1.25 | m |
| YQ130 | 1 | 11.98+5.77=17.75 | 2.08 | sm | YQ131 | 1 | 11.86+7.68=19.54 | 1.54 | sm |
|  | 2 | 8.37+5.59=13.96 | 1.50 | m |  | 2 | 7.72+5.81=13.53 | 1.33 | m |
|  | 3 | 7.82+5.07=12.89 | 1.54 | m |  | 3 | 7.22+5.22=12.44 | 1.38 | m |
|  | 4 | 7.59+5.17=12.76 | 1.47 | m |  | 4 | 6.70+5.34=12.04 | 1.25 | m |
|  | 5 | 7.11+4.81=11.93 | 1.48 | m |  | 5 | 7.24+4.35=11.59 | 1.67 | m |
|  | 6 | 6.27+5.00=11.27 | 1.25 | m |  | 6 | 6.47+4.87=11.34 | 1.33 | m |
|  | 7 | 6.07+4.76=10.83 | 1.28 | m |  | 7 | 5.93+4.76=10.69 | 1.25 | m |
|  | 8 | 4.50+4.10=8.61 | 1.10 | m |  | 8 | 4.89+3.94=8.82 | 1.24 | m |
| HZ139 | 1 | 10.98+8.88=19.85 | 1.24 | m | HZ144 | 1 | 11.21+7.30=18.52 | 1.54 | m |
|  | 2 | 7.65+6.10=13.74 | 1.25 | m |  | 2 | 8.04+5.78=13.81 | 1.39 | m |
|  | 3 | 6.82+5.92=12.74 | 1.15 | m |  | 3 | 7.05+5.75=12.80 | 1.23 | m |
|  | 4 | 6.63+5.04=11.66 | 1.32 | m |  | 4 | 7.55+4.70=12.25 | 1.61 | m |
|  | 5 | 6.56+4.74=11.30 | 1.39 | m |  | 5 | 7.00+5.01=12.01 | 1.40 | m |
|  | 6 | 5.72+4.82=10.54 | 1.19 | m |  | 6 | 6.57+4.70=11.28 | 1.40 | m |
|  | 7 | 5.66+4.60=10.26 | 1.23 | m |  | 7 | 5.81+4.50=10.31 | 1.29 | m |
|  | 8 | 5.66+4.23=9.89 | 1.34 | m |  | 8 | 5.39+3.63=9.02 | 1.49 | m |
| HZ145 | 1 | 11.30+8.64=19.94 | 1.31 | m | HZ152 | 1 | 13.29+6.10=19.39 | 2.18 | sm |
|  | 2 | 8.88+5.44=14.32 | 1.63 | m |  | 2 | 9.86+5.24=15.10 | 1.88 | sm |
|  | 3 | 7.85+5.10=12.95 | 1.54 | m |  | 3 | 8.26+5.16=13.42 | 1.60 | m |
|  | 4 | 6.96+5.69=12.65 | 1.22 | m |  | 4 | 7.74+5.13=12.86 | 1.51 | m |
|  | 5 | 6.69+4.89=11.58 | 1.37 | m |  | 5 | 6.64+4.61=11.25 | 1.44 | m |
|  | 6 | 6.61+4.00=10.61 | 1.66 | m |  | 6 | 6.33+4.47=10.80 | 1.42 | m |
|  | 7 | 5.61+4.18=9.79 | 1.34 | m |  | 7 | 5.52+4.33=9.85 | 1.28 | m |
|  | 8 | 4.72+3.45=8.17 | 1.37 | m |  | 8 | 3.96+3.36=7.33 | 1.18 | m |
| RHC157 | 1 | 13.00+5.88=18.88 | 2.21 | sm | RHC158 | 1 | 11.33+6.98=18.32 | 1.62 | m |
|  | 2 | 9.23+6.32=15.55 | 1.46 | m |  | 2 | 8.35+5.94=14.29 | 1.41 | m |
|  | 3 | 7.41+6.13=13.54 | 1.21 | m |  | 3 | 8.09+4.79=12.88 | 1.69 | m |
|  | 4 | 7.04+5.17=12.21 | 1.36 | m |  | 4 | 7.34+4.57=11.90 | 1.61 | m |
|  | 5 | 6.34+4.92=11.25 | 1.29 | m |  | 5 | 6.35+5.09=11.45 | 1.25 | m |
|  | 6 | 6.23+4.47=10.70 | 1.39 | m |  | 6 | 6.27+4.65=10.92 | 1.35 | m |
|  | 7 | 5.29+4.01=9.30 | 1.32 | m |  | 7 | 5.59+4.57=10.16 | 1.22 | m |
|  | 8 | 4.75+3.80=8.56 | 1.25 | m |  | 8 | 5.69+4.39=10.09 | 1.30 | m |
| CPC159 | 1 | 10.90+7.70=18.60 | 1.42 | m | WJZ5 | 1 | 9.77+7.10=16.88 | 1.38 | m |
|  | 2 | 6.86+6.28=13.15 | 1.09 | m |  | 2 | 7.59+5.88=13.47 | 1.29 | m |
|  | 3 | 7.03+5.45=12.48 | 1.29 | m |  | 3 | 7.11+5.96=13.07 | 1.19 | m |
|  | 4 | 6.84+5.72=12.56 | 1.20 | m |  | 4 | 7.10+5.81=12.91 | 1.22 | m |
|  | 5 | 6.38+5.14=11.53 | 1.24 | m |  | 5 | 6.48+5.54=12.02 | 1.17 | m |
|  | 6 | 6.22+4.45=10.67 | 1.40 | m |  | 6 | 6.09+5.25=11.34 | 1.16 | m |
|  | 7 | 5.93+4.73=10.66 | 1.25 | m |  | 7 | 5.58+5.09=10.66 | 1.10 | m |
|  | 8 | 6.00+4.36=10.36 | 1.38 | m |  | 8 | 5.07+4.58=9.66 | 1.11 | m |
| ZZ12 | 1 | 12.40+5.57=17.97 | 2.23 | sm | LCC16 | 1 | 10.8+6.20=17.04 | 1.75 | sm |
|  | 2 | 9.60+5.38=14.98 | 1.78 | sm |  | 2 | 8.42+5.85=14.26 | 1.44 | m |
|  | 3 | 9.28+5.24=14.51 | 1.77 | sm |  | 3 | 7.90+5.94=13.84 | 1.33 | m |
|  | 4 | 7.22+5.22=12.44 | 1.38 | m |  | 4 | 7.34+5.10=12.44 | 1.44 | m |
|  | 5 | 7.51+4.64=12.15 | 1.62 | m |  | 5 | 7.25+5.01=12.26 | 1.45 | m |
|  | 6 | 6.56+4.75=11.31 | 1.38 | m |  | 6 | 6.37+4.76=11.12 | 1.34 | m |
|  | 7 | 5.00+4.28=9.28 | 1.17 | m |  | 7 | 6.09+4.30=10.39 | 1.42 | m |
|  | 8 | 3.83+3.51=7.34 | 1.09 | m |  | 8 | 4.89+3.76=8.65 | 1.30 | m |
| XCX23 | 1 | 11.51+9.86=21.37 | 1.17 | m | DJPC31 | 1 | 10.91+7.21=18.11 | 1.51 | m |
|  | 2 | 8.03+5.85=13.88 | 1.37 | m |  | 2 | 8.00+5.64=13.64 | 1.42 | m |
|  | 3 | 6.93+5.55=12.48 | 1.25 | m |  | 3 | 6.93+5.93=12.87 | 1.17 | m |
|  | 4 | 6.90+5.42=12.33 | 1.27 | m |  | 4 | 6.69+5.65=12.34 | 1.18 | m |
|  | 5 | 6.70+4.69=11.39 | 1.43 | m |  | 5 | 6.51+5.40=11.91 | 1.21 | m |
|  | 6 | 5.92+4.86=10.79 | 1.22 | m |  | 6 | 6.20+5.30=11.51 | 1.17 | m |
|  | 7 | 5.19+4.18=9.36 | 1.24 | m |  | 7 | 5.32+4.82=10.14 | 1.10 | m |
|  | 8 | 4.61+3.79=8.41 | 1.22 | m |  | 8 | 5.14+4.34=9.48 | 1.18 | m |

**Note:** EMN: Experimental material name; CN: Chromosome numbering; RL: Relative length, m: metacentric; sm: submetacentric.
